# Circulating tumor cells in peripheral and pulmonary venous blood predict poor long-term survival in resected non-small cell lung cancer patients

**DOI:** 10.1038/s41598-017-05154-x

**Published:** 2017-07-10

**Authors:** Yunsong Li, Xu Cheng, Zhong Chen, Yi Liu, Zhidong Liu, Shaofa Xu

**Affiliations:** 1 No.2 Department of Chest Surgery, Beijing Chest Hospital, Capital Medical University/Beijing Tuberculosis and Thoracic Tumor Research Institute, Beijing, China; 2 Head and Neck Surgery Branch NIDCD/NIH 10 Center Drive, Rm 5D5, Bethesda, MD 20892 USA; 3 Department of Bacteriology and Immunology, Beijing Key Laboratory on Drug-Resistant Tuberculosis Research, Beijing Tuberculosis and Thoracic Tumor Research Institute/Beijing Chest Hospital, Capital Medical University, Tongzhou District, Beijing 101149 PR China

## Abstract

We tested the hypothesis that circulating tumor cells (CTCs) in preoperative peripheral blood (PPB) and intraoperative pulmonary venous blood (IPVB) could predict poor long-term survival in resected non-small cell lung cancer (NSCLC) patients. CTCs were separated from blood using magnetic beads coated with antibodies against epithelial-cell adhesion molecule (EpCAM) via magnetic-activated cell sorting (MACS). CTCs were quantified with fluorescence-labeled antibodies against pan-cytokeratin through flow cytometry. CTCs were quantified in PPB and IPVB in 23 consecutive stage I-IIIA patients with resected NSCLC. The association between CTCs and prognosis in these patients was evaluated after a 5-year follow-up. In NSCLC patients, outcomes were assessed according to CTC levels at surgery. NSCLC patients identified as high-risk groups exhibited >5 CTCs/15 mL in PPB and >50 CTCs/15 mL in IPVB. Univariate Cox proportional-hazards regression analysis showed that the CTC count in PPB or IPVB was an independent risk factor for tumor-free surivival (TFS) and overall survival (OS). The high-risk group of patients had a shorter median TFS (22 months vs. >60.0 months, *p* < 0.0012) and shorter OS (27 months vs. >60 months, *p* < 0.0015). The number of CTCs counted in PPB and IPVB was an independent risk factor for TFS and OS in resected NSCLC patients.

## Introduction

Non-small cell lung cancer (NSCLC) is one of the most aggressive malignant tumors with the highest morbidity and mortality worldwide. Surgical resection is the preferred comprehensive treatment for stage I-IIIA NSCLC; however, postoperative metastases and recurrence still occur in early-stage cancer patients, even reaching approximately 29% in stage I patients^1^. Early-stage NSCLC patients have different prognoses, and the progression of disease is primarily due to micro- or occult metastasis and recurrent tumors^2^. After surgery, no clinical imaging or other technology can sensitively detect early and micro- or occult metastases in these lung cancer patients^3, 4^. Once metastasis or recurrent cancer are clinically observed, the tumors are quite large and difficult to treat, and the patients have lost the best time window for effective therapy. During postoperative follow-up, a more sensitive and reliable detection method is needed to detect the early warning signs of occult metastasis and recurrence to guide individualized therapies. Recent technological advances regarding the detection of circulating tumor cells (CTCs) in the blood of cancer patients could fill the gap in clinical diagnostics.

According to the “seed and soil” theory, CTCs may be the “seed cells” leading to distant metastasis. CTCs are tumor cells that have broken free from the main tumor and entered the bloodstream^5, 6^. CTCs may have access to favorable distant sites where they can implant themselves to grow into a metastasis^7, 8^. Thus far, most studies on CTCs have focused on patients with stage IV cancers^9^, while the clinical significance of CTCs in the early stage of surgically resected cancer patients has rarely been investigated^10^.

Here, we evaluated CTCs in peripheral and pulmonary venous blood from stage I-IIIA postoperative NSCLC patients beginning in 2010^11, 12^. We developed a CTC detection technique using magnetic-activated cell sorting (MACS) and flow cytometry, termed the FAMCell System. A 5-year follow-up study was conducted to identify the association of long-term survival with CTC counts in these patients to establish a clinical diagnostic intervention for future large-scale clinical trials^13^.

## Results

### Patient characteristics

The present study included 23 consecutive NSCLC patients who underwent complete resection between March and April in 2010 and 5-year postoperative follow-up (Table 1). The average (±SD) age of the patients was 61.7 ± 9.5 years (median, 61.7); 52.2% of the patients presented with squamous carcinoma, 47.8% of the patients presented with adenocarcinoma, and 26.1% of the patients presented with lymph-node metastasis.

**Table 1:** Surgically Resected Non-Small Cell Lung Cancer Patients Characteristics.

Items	Evaluable Patients (n = 23)	
	No.	%
Age (years)		
Median	61.7	
Range	43–78	
Sex		
Male	8	34.8
Female	15	65.2
Pathological type		
Squamous carcinoma	12	52.2
Adenocarcinoma	11	47.8
Pathological stage		
I	8	34.8
II	7	30.4
IIIA	8	34.8
Lymph-node metastasis^‡^		
Positive	6	26.1
Negative	17	73.9
Smoking index^§^		
≥400	13	56.5
<400	10	43.5
Tumor free survival(months)		
Mean	42.0	
Range	1–60^*^	
Overall survival(months)		
Mean	43.6	
Range	5–60^*^	

^‡^Cancer patients were pathologically confirmed and divided into lymph node metastasis positive (N1–N3), or negative (N0), respectively.

^§^Smoking index = daily cigarette number × year of smoking; Smoking index ≥400 is a high-risk factor of lung cancer.

^*^The last follow-up of tumor free survival or overall survival was 60 months.

By the end of the 5-year follow-up, the mean tumor-free survival (TFS) and overall survival (OS) of the 23 patients were 42.0 ± 22.5 and 43.6 ± 21.0 months, respectively. A total of thirteen patients (56.5%) survived, while 10 patients (43.5%) exhibited progressive disease development and death. The 5-year survival rates of the patients with stage I, II, and IIIA NSCLC were 87.5% (7/8), 71.4% (5/7), and 12.5% (1/8), respectively.

### CTC quantification and positive cutoff value

**Figure 1:**
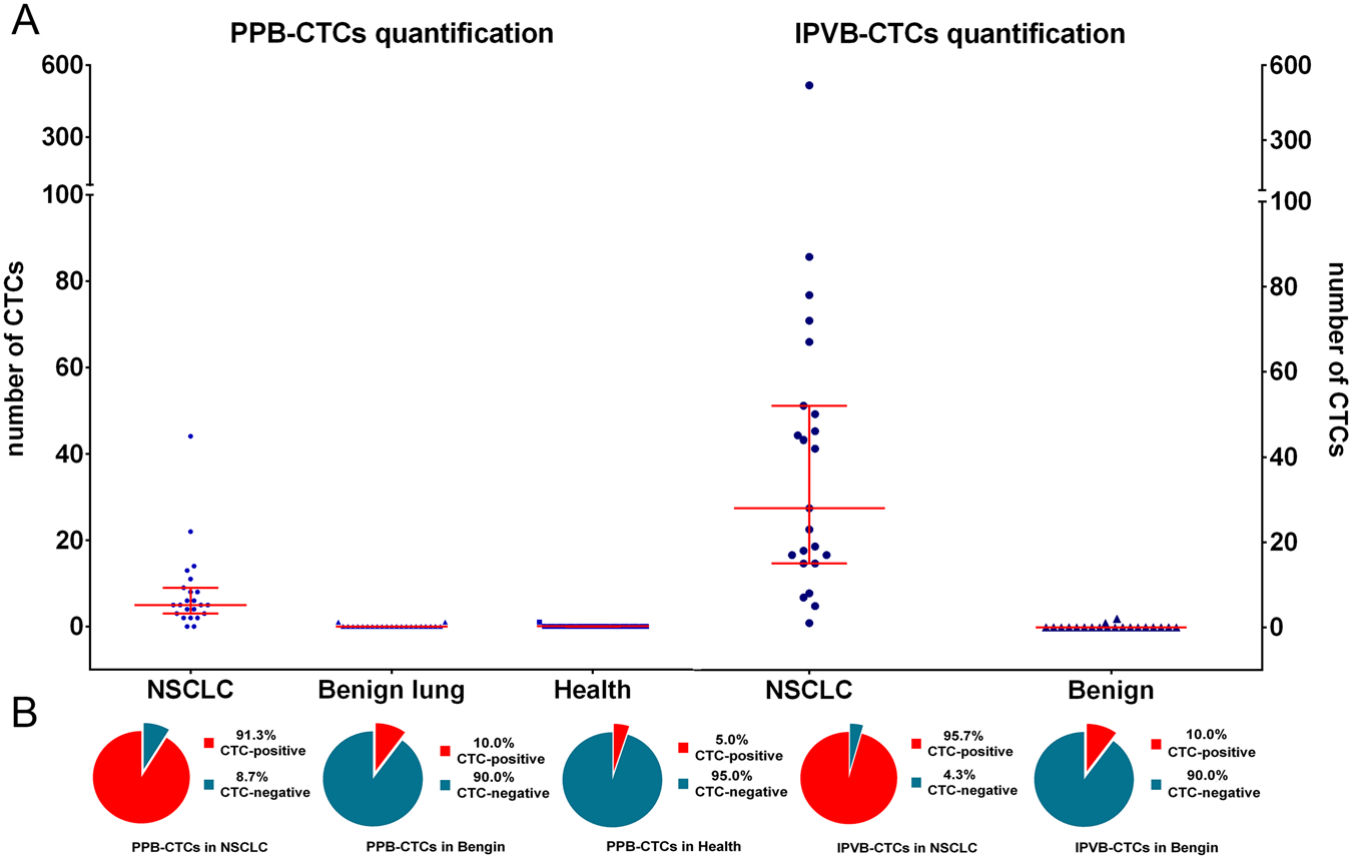
Analysis of circulating tumor cells in different groups. (**A**) Scatter plots showing the number of CTCs in different groups drawn using blue points, and the median and interquartile range are indicated by red lines. (**B**) Pie chart showing the ratio of CTC-positive and CTC-negative patients in different groups. Abbreviations: *CTCs: circulating tumor cells; PPB: preoperative peripheral blood; IPVB: intraoperative pulmonary venous blood; NSCLC: non-small cell lung cancer; Benign: benign lung disease group; Health: health volunteers group.

CTCs were isolated and quantified from two blood sources (PPB and IPVB) of NSCLC patients, benign lung disease patients, and healthy volunteers using the FAMCell System (Fig. 1). In the PPB samples, CTC-positive specimens were detected in 2/20 (10%) patients with benign pulmonary disease (median 0, range 0–1), and in 1/20 (5%) healthy volunteers (median 0, range 0–1). In the IPVB samples, CTC-positive specimens were detected in 2/20 (10%) patients with benign pulmonary disease (median 0, range 0–1). The range of CTC counts in three control groups were all ≤1.0 CTCs/15 mL. Thus, the CTC positive cutoff value was set to ≥2.0 CTCs/15 mL, and this cutoff could be used as a diagnostic indicator in NSCLC patients, *p* < 0.001. The median PPB CTCs was 5/15 mL (interquartile range, 3–9), which was significantly lower than the corresponding median IPVB CTCs of 28/15 mL (interquartile range, 15–52; *p* < 0.001). In the PPB specimens of the NSCLC group, CTC-positive specimens were detected in 21/23 patients, corresponding to a CTC-positive rate of 91.3%. In the IPVB specimens of the NSCLC group, CTC-positive specimens were detected in 22/23 patients, corresponding to a CTC-positive rate of 95.7%.

### Prevalence and association of PPB-CTCs and IPVB-CTCs high-risk cutoff values with clinical features

We next investigated the prognosis value of CTCs for metastasis and recurrence in the high-risk patient group. The best high-risk cutoff value that yielded the largest sum of sensitivity and specificity based on ROC curve analysis together with the cutoff points correspond to the largest hazard ratio (HR) according to the univariate Cox regression analysis (Supplemental Figures S2 and S3). CTC counts of >5/15 mL in PPB and >25/15 mL in IPVB were selected as the best high-risk cutoff values for significantly detecting survival. Patients with CTC counts above and below the high-risk cutoff values were divided into high/low-risk groups. The high-risk rates of CTCs in the PPB and IPVB specimens were 43.5% and 26.1%, respectively, in the NSCLC group (Fig. 1).

The prevalence of CTCs in PPB/IPVB and its association with clinical characteristics in the NSCLC group are listed in Table 2. In PPB and IPVB specimens, the CTC high-risk rate was significantly correlated with the pathological stage of NSCLC (PPB-CTCs: *p* < 0.0001, and IPVB-CTCs *p* = 0.013), N stage (PPB-CTCs: *p* = 0.002, and IPVB-CTCs: *p* = 0.021) and disease status (PPB-CTCs: *p* = 0.003, and IPVB-CTCs: *p* = 0.002).

**Table 2:** Prevalence of circulating tumor cells and association with clinical features (n = 23)*.

Variable	Patients with CTCs in PPB		Patients with CTCs in IPVB	
	>5 (n = 10)	≤5 (n = 13)	>50 (n = 6)	≤50 (n = 17)
Sex				
Male (n = 15)	6	9	4	11
Female (n = 8)	4	4	2	6
Fisher’s exact *P*	0.685		>0.99	
Pathological type				
Squamous carcinoma (n = 12)	3	9	1	11
Adenocarcinoma (n = 11)	7	4	5	6
Fisher’s exact *P*	0.100		0.069	
Pathological stage				
I (n = 8)	1	7	1	7
II (n = 7)	1	6	0	7
IIIA (n = 8)	8	0	5	3
Pearson Chi-square *P*	0.000		0.013	
T stage				
1 (5)	1	4	0	5
2 (12)	6	6	4	8
3 (3)	0	3	0	3
4 (3)	3	0	2	1
Pearson Chi-square *P*	0.057		0.126	
N stage				
N0 (17)	4	13	2	15
N1-N3 (6)	6	0	4	2
Fisher’s exact *P*	0.002		0.021	
Smoking index				
≥400(n = 13)	5	8	3	10
<400(n = 10)	5	5	3	7
Mcnemar test *P*	0.686		>0.99	
Disease status†				
Progress (n = 10)	8	2	6	4
Stable (n = 13)	2	11	0	13
Fisher’s exact *P*	0.003		0.002	

^*^CTCs circulating tumor cells, PPB preoperative peripheral blood, IPVB-CTCs intraoperative pulmonary venous blood.

^†^Progression was defined according to the Response Evaluation Criteria in Solid Tumors. It meant metastases and recurrence occurring after surgical resection.

### Prognostic significance of CTC count at baseline

**Figure 2:**
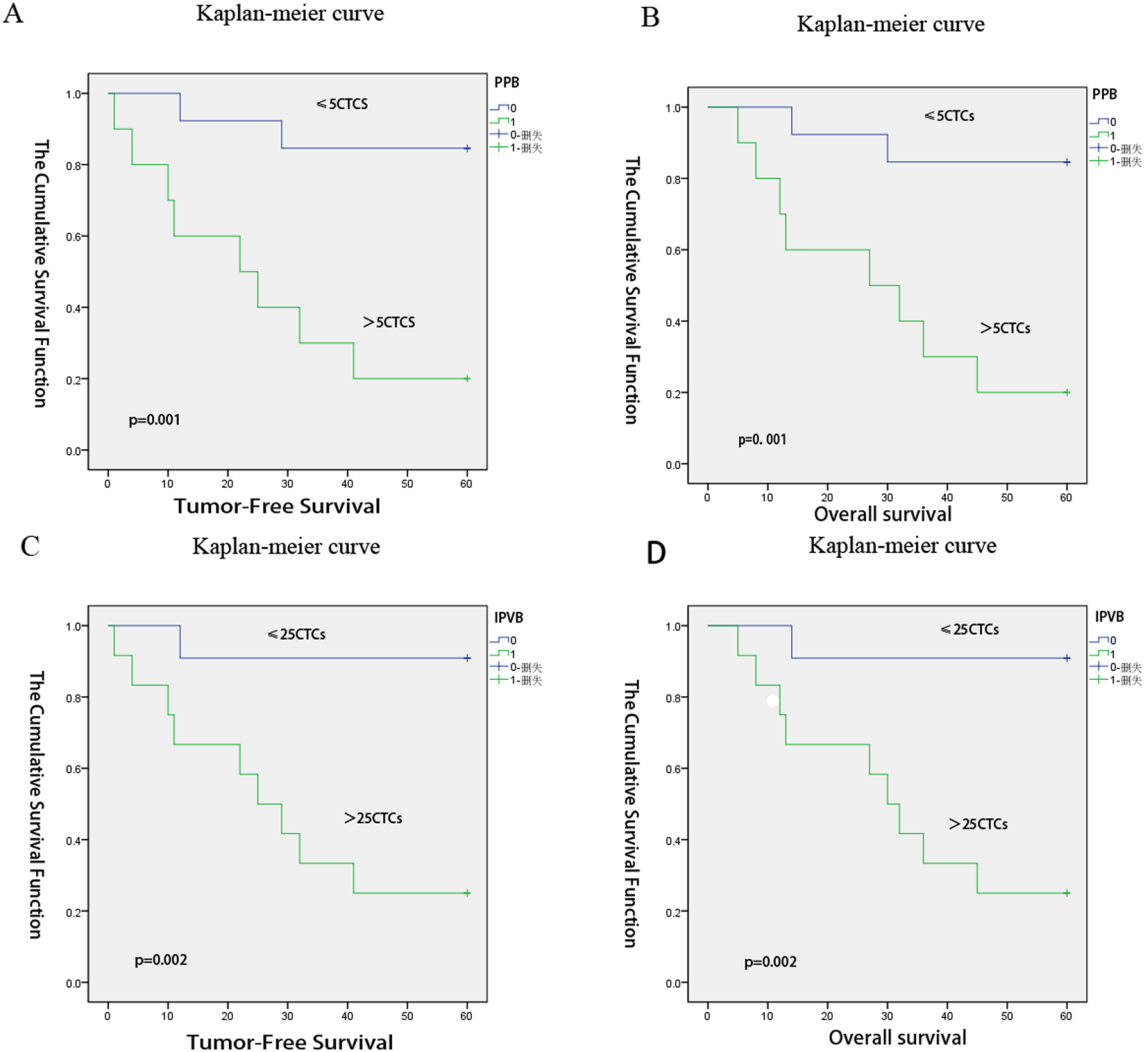
Kaplan–Meier estimates of probabilities of progression-free survival and overall survival in patients with non-small cell lung cancer at the follow-up times*. As shown in panels (A,B,C and D), the follow-up time for the study was 5 years. We defined NSCLC patients with PPB-CTC density >5 CTCs/15 mL as the high-risk group and those with ≤5 CTCs/15 mL as the low-risk group. Panel A shows the probability of TFS according to the PPB-CTCs; the median TFS was 22.0 months for high-risk patients and >60 months for low-risk patients, *p* = 0.001 via the log-rank test; Panel B shows the probability of OS according to the PPB-CTCs; the median was 27.0 months for high-risk patients and >60 months for low-risk patients, *p* = 0.001 via the log-rank test. Using the IPVB specimens, we defined the patients with IPVB-CTC density > 25 CTC/15 mL as the high-risk group and those with IPVB-CTC density ≤25 CTCs/15 mL as the low-risk group. Panel C shows the probability of TFS according to the IPVB-CTCs; the median was 25.0 months for high-risk patients and >60.0 months for low-risk patients, *p* = 0.002 via the log-rank test. Panel D shows the probability of OS according to the IPVB-CTCs; the median was 30.0 months for high-risk patients and >60.0 months for low-risk patients, p = 0.002 via the log-rank test. Abbreviations: TFS: tumor-free survival; OS: over-survival; PPB-CTCs: peripheral blood circulating tumor cells; IPVB-CTCs: pulmonary venous blood circulating tumor cells.

In the Kaplan-Meier survival analysis and univariate Cox regression analysis of CTC counts, we defined NSCLC patients with PPB-CTC density >5 CTCs/15 mL as the high-risk group and those with ≤5 CTCs/15 mL as the low-risk group. The median TFS was 22.0 months for the high-risk group and >60 months for the low-risk group according to the log-rank test (*p* = 0.001) (Fig. 2) and the Cox proportional hazards regression (HR, 8.71, *p* = 0.007) (Table 3). The median OS was 27.0 months for high-risk patients and >60 months for low-risk patients according to the log-rank test (*p* = 0.001) (Fig. 2B) and Cox proportional hazards regression (HR, 8.41, *p* = 0.007).

**Table 3:** Univariate Cox regression analysis for prediction of tumor-free survival and overall-survival*.

Parameter	At-risk group	TFS risk		OS risk	
	High vs. Low	*P*	HR	*P*	HR
TNM Stage	IIIA vs. II vs. I	0.004	4.48	0.005	4.32
N stage	N1–2 vs. N0	0.026	4.17	0.031	3.98
PPB-CTCs	>5 vs. ≤5	0.007	8.71	0.007	8.41
IPVB-CTCs	>25 vs. ≤25	0.016	12.88	0.016	12.88

^*^Abbreviations: TFS: tumor-free survival and OS: overall-survival. HR: hazard ratio; PPB-CTCs: peripheral blood circulating tumor cells; IPVB-CTCs: pulmonary venous blood circulating tumor cells.

Using the PPB specimens, results showed that the TFS, Cox proportional hazards regression HR, 8.71; *P* = 0.007; the OS, Cox proportional hazards regression HR, 8.71; *P* = 0.007. Using the IPVB specimens, results showed that the TFS, Cox proportional hazards regression HR, 12.88; *P* = 0.0162, whereas the OS, Cox proportional hazards regression HR, 12.88; *P* = 0.016.

Using the IPVB specimens, we defined patients with IPVB-CTC density >25 CTC/15 mL as the high-risk group and those with IPVB-CTC density ≤25 CTCs/15 mL as the low-risk group. The median TFS was 25.0 months for high-risk patients and >60.0 months for low-risk patients according to the log-rank test (*p* = 0.002) (Fig. 2C) and Cox proportional hazards regression (HR, 12.882, *p* = 0.016) (Table 3). The median OS was 30.0 months for high-risk patients and >60.0 months for low-risk patients according to the log-rank test (*p* = 0.002) (Fig. 2D) and Cox proportional hazards regression (HR, 12.882, *p* = 0.016).

## Discussion

In this study, we tested the hypothesis that tumor cells of stage I-IIIA NSCLC patients were able to spread to the entire body via pulmonary and peripheral venous blood vessels and identified the relationship between CTCs and poor long-term survival in surgically resected NSCLC patients. It is widely accepted that metastasis is a late event in cancer progression. However, we show in this study that tumor cells can disseminate systemically from stage I-IIIA NSCLC surgically resected patients. CTCs in pulmonary venous vessels can spread to the systemic circulation via the aorta and then back to the pulmonary circulation from peripheral venous vessels. Thus, simultaneously identifying CTCs in both PPB and IPVB in one patient could be a strong indication that tumor cells are spreading throughout the entire body. Our clinical investigation in this study supports this hypothesis, as we observed that CTCs were positive in both PPB and IPVB in 87.0% of patients, which included 7 patients with stage I tumors. Furthermore, owing to the difficulty obtaining blood samples from surgery, few studies have reported CTCs identified in IPVB^14^. Our observations are consistent with other reports that CTCs can be detected in PPB and IPVB at comparable sensitivities from the early stage of cancer patients, such as in prostate cancer, breast cancer, NSCLC, pancreatic cancer and melanoma^15–18^.

Using current image technologies, the size of NSCLC tumors required for accurate diagnosis is larger than 1 cm in diameter. It is a clinical challenge to diagnose occult lung tumor foci smaller than 1 cm in diameter. The primary complication for lung cancer patients after surgery is distance metastasis; when metastasis occurs, the 1-year survival rate is only approximately 20–23%^19^. As there is no imaging technology capable of evaluating smaller lung cancer nodules and early metastasis, these patients miss the opportunity for treatment. In this study, of the 10 patients in which metastasis occurred, the mean time from metastasis to death was only 3.2 ± 2.7 months, and mean TFS was 19.0 ± 12.7 months. Thus, an early-warning index of occult metastasis for personal individualized therapeutics is highly needed, and the results demonstrated here using CTC technology could fill the gap in clinical diagnostics.

The control group included 20 patients with benign lung diseases who underwent surgery and 20 healthy volunteers. There are 4 cases of bronchiectasis and 7 cases of tuberculosis who simultaneously suffered from severe pulmonary infection. However, the CTC results should not be influenced by pulmonary infection or post-obstructive pneumonitis.

In this study, we found that the high-risk rate of patients as detected by CTCs was significantly associated with N staging (PPB: *p* = 0.002, IPVB: *p* = 0.021), but a significant association with T staging was not found, likely due to the small sample size (PPB: *p* = 0.057, IPVB: *p* = 0.126). This result suggests that CTCs are more significantly associated with the metastatic capacity of NSCLC foci than with tumor sizes, which is consistent with studies of head and neck squamous cell carcinomas and breast cancer^20, 21^. Furthermore, CTCs can be detected at the precancerous stage, which could benefit patients by allowing early cancer detection and monitoring of disease progression^22^. After increasing the sensitivity and reproducibility of the CTC testing method, CTCs could be used to detect distant metastasis before tumor foci was detectable by imaging technology. Thus, CTC technology could be used in the clinic as an indicator of occult metastasis.

In this study, the most widely known epithelial phenotype marker epithelial-cell adhesion molecule (EpCAM) and pan-cytokeratin(CK) were used to separate and detect CTCs. Although we could not detect EMT phenotype CTCs, the results of metastatic malignancy support that epithelial phenotype CTCs are a risk factor the curative effect and prognosis of patients. In addition, there is currently no recognized EMT phenotype marker. The PPB and IPVB cutoff points were analyzed by ROC curve analysis. Although this study is based on a small population of patients, the analysis procedure is precise, and the results are highly credible. We have shown that PPB-CTCs >5/15 mL or IPVB-CTCs > 25/15 mL can be used as independent prognostic factors of surgically resected NSCLC patients for a 5-year follow-up^23, 24^. In advanced lung cancer patients, CTCs ≥ 5/ml in PPB is also an independent prognostic factor. The development of distant metastasis depends on the ability of the tumor immunogenicity to escape immune surveillance and on the proliferation potential of CTCs^25–27^. Therefore, dormant CTCs could be a “silent bomb” within the bodies of lung cancer patients^28^. Regarding tumor-free survival of these patients, the occurrence of metastasis or recurrence depends on the balance of tumor aggressiveness and anti-tumoral activities, including immune surveillance and postoperative adjuvant therapy. Although the primary tumor (T) and mediastinal lymph node metastasis (N) are resected during surgery, distant metastases (M1) have not yet developed, and therefore, CTCs are the only therapeutic indicator that can be detected at this stage. It has been reported that chemotherapy can decrease peripheral blood CTCs in advanced tumor patients. We are currently in the process of developing personalized interventions for tumor-free survival of NSCLC patients with high-risk CTCs based on CTC detection.

## Materials and Methods

### Study subjects

The present prospective study was carried out at the Beijing Chest Hospital affiliated with Capital Medical University (Beijing, China). Patients were enrolled from March to April 2010. The inclusion criteria for NSCLC patients were as follows: 1) NSCLC cTNM stage I-IIIA; 2) initial surgical treatment for NSCLC by complete resection at our hospital; and 3) Eastern Cooperative Oncology Group (ECOG) scores of 0–2 for performance status. The inclusion criterion for benign lung diseases patients was diagnosis of a benign lung disease requiring surgery. The inclusion criterion for healthy volunteers was simply to be healthy. The exclusion criterion for all subjects was a history of anti-cancer therapy. Twenty-three patients were included in the NSCLC group. The control group included 20 patients with benign lung diseases undergoing surgery (3 cases of hamartoma tumor, 4 cases of bronchiectasis, and 13 cases of tuberculosis) and 20 healthy volunteers. The study was approved by the Institutional Review Board of the Beijing Chest Hospital, Capital Medical University, and written informed consent was obtained from all patients.

### Blood sampling

NSCLC patients were treated via complete resection according to the 2007 National Comprehensive Cancer Network (NCCN) NSCLC Guidelines. PPB specimens were taken from the median cubital vein prior to surgery, and IPVB specimens were taken before blocking the pulmonary vein during surgery. In the benign lung disease group, both peripheral and pulmonary vein blood was sampled. In the healthy volunteer group, only peripheral blood was taken. For each sample, 15-mL blood was stored in three 5-mL EDTA-K_3_-containing blood collection tubes (Greiner Bio-One, Kremsmunster, Austria). All tests were completed within 12 hours after collection.

### CTC sorting by AutoMACS, optimization of flow cytometry conditions, and CTCs quantitative detection

The FAMCell System separates and enumerates epithelial CTCs from the blood using anti-EpCAM antibody-coated magnetic beads and then identifies pan-cytokeratin-positive and CD45-negative cells using fluorescently labeled antibodies against pan-cytokeratin and CD45. The FAMCell System was used to quantitatively detect CTCs in blood specimens from NSCLC surgically resected patients. Detailed information is presented in the Supplemental Materials and Methods.

### Follow-up

Postoperative adjuvant treatment and follow-up were carried out strictly according to the NCCN 2007 NSCLC Guidelines^29^. The study was a blinded, single-center imaging study for evaluating objective treatment responses and disease progression. Imaging evaluation was performed for all patients during the follow-up by an independent radiologist according to the Response Evaluation Criteria in Solid Tumors^30^.

### Statistical analysis

Data were analyzed using SPSS 20.0 (SPSS Inc., Chicago, IL, USA). Paired t-tests were used to evaluate the variation in the CTC counts in preoperative peripheral blood (PPB-CTCs) and in intraoperative pulmonary venous blood (IPVB-CTCs). Receiver operating characteristics (ROC) curve analysis and the univariate Cox proportional-hazards regression model were utilized to determine the optimal high-risk cutoff values for PPB-CTC and IPVB-CTC specimens. Accordingly, NSCLC patients were divided into high/low-risk groups, and Fisher’s exact tests were utilized to evaluate the differences in their clinical features. In the survival analysis, TFS was defined as the period between the end of surgery and cancer recurrence or metastasis. OS was defined as the period between the end of surgery and death or the last contact. Log-rank tests were utilized to examine differences in survival. The univariate Cox proportional-hazards regression model was utilized to evaluate the hazard ratios of TFS and OS.

## Electronic supplementary material

Supplementary Appendix
