# Supplementary material for: Circulating tumor cells in peripheral and pulmonary venous blood predict poor long-term survival in resected non-small cell lung cancer patients
Source: Sci Rep. 2017 Jul 10;7:4971. doi: 10.1038/s41598-017-05154-x (PMC5503943; doi:10.1038/s41598-017-05154-x)
Supplement: Supplementary file 1 — Supplementary Appendix [file 41598_2017_5154_MOESM1_ESM.pdf]

Circulating tumor cells in peripheral and pulmonary venous blood predict poor long-term survival in resected non-small cell lung cancer patients

Yunsong Li[1†], Xu Cheng[1†], Zhong Chen[2],Yi Liu, Zhidong Liu[1*], Shaofa Xu[1*]

[1]No.2 Department of Chest Surgery, Beijing Chest Hospital, Capital Medical University/Beijing Tuberculosis and Thoracic Tumor Research Institute, Beijing, China

[2]Head and Neck Surgery Branch NIDCD/NIH 10 Center Drive, Rm 5D5, Bethesda, MD 20892, USA

†Those authors contributed equally to this work

***Corresponding author**

Dongzhi Liu

No.2 Department of Chest Surgery, Beijing Chest Hospital, Capital Medical University/Beijing Tuberculosis and Thoracic Tumor Research Institute, Beijing, China

Tel: +86-13691297853

Fax: +86-021-64085875

E-mail: liuzhidong@ccmu.edu.cn

Shaofa Xu

No.2 Department of Chest Surgery, Beijing Chest Hospital, Capital Medical University/Beijing Tuberculosis and Thoracic Tumor Research Institute, Beijing, China

Tel: +86-13691297853

Fax: +86-021-64085875

E-mail: xushaofa_1@126.com

Running title: Circulating tumor cells in surgically resected NSCLC

Supplementary Appendix

This appendix is provided by the authors to provide more information about the method used to quantify circulating tumor cells (CTCs).

Appendix A: CTC sorting by AutoMACS and optimization of flow cytometry conditions and CTC quantitative detection

Part 1: CTC sorting by AutoMACS

Separation of CTCs was achieved using an automatic magnetic cell sorter (AutoMACS) (Miltenyi Biotec, Bergisch Gladbach, Germany). Epithelial cells were marked and separated with immunomagnetic beads coated with EpCAM using the AutoMACS and the POSSELDs program. Tumor cells were then enriched 104 times.

Positive and negative sorting strategies were compared using the AutoMACS, and the recovery was higher with positive sorting ($p<0.05$). We considered that intercellular non-specific adhesion between tumor cells and leukocytes could lead to the loss of tumor cells during negative sorting. The purity was also higher with positive sorting, as there were more impurities in the negative-sorted samples. Therefore, we selected the positive sorting strategy. When using positive sorting, the POSSELD program may be repeated twice (POSSELDs). Indeed, the POSSELD program can enrich tumor cells by 102 times with a recovery rate of approximately 80%, while the POSSELDs program can enrich tumor cells by 104 times with a recovery rate of approximately 64%. Thus, we selected the POSSELDs program to increase the enrichment of tumor cells.

Part 2: Optimization of flow cytometry conditions and CTC quantitative detection

After enrichment of epithelial cells, quantitative detection of CTCs was carried out using flow cytometry [1-4]. Fluorescence-labeled monoclonal antibodies against

leukocytes (CD45-phycoerythrin) and epithelial cells (cytokeratins 7-, 8-, 18-, and 19-fluorescein isothiocyanate) were used to distinguish epithelial cells from leukocytes using a FACSCalibur flow cytometer (BD Biosciences, Franklin Lake, NJ, USA) and CellQuest software (BD Biosciences, Franklin Lake, NJ, USA). CTCs were defined as cells lacking CD45 and expressing cytokeratins 7, 8, 18 and 19. The gating strategy of the flow cytometer is shown in Figure S1A.

Three controls were selected to eliminate false-positive cells and to control false-negative results. 1) Inner control: The POSSELDs program of the AutoMACS was used to enrich EpCAM-positive tumor cells by 104 times. For each NSCLC patient, we used a specimen from the sample before submitting the sample to the POSSELDs program as the inner control. The final result of each sample was the number of EpCAM-positive cells minus the inner control. 2) Normal control: The normal control group included 20 patients with benign lung diseases who underwent surgery and 20 healthy volunteers. 3) Positive control: The LTEP-A2 human lung cancer cells were spiked into blood samples from healthy volunteers as a positive control. This control was used to optimize the flow cytometry conditions.

Appendix B

Figure S1. The gating strategy of flow cytometry and CTCs in patient No.3

Figure S1 legend

Panel A (left) shows the gating strategy of G1. G1=R1. Gate 1 was assigned to the unlysed erythrocytes and cell debris. By taking cells located outside region 1 (i.e., not region 1), we avoided interference from erythrocytes and cell debris. Panel A (right) shows the gating strategy of G2. G2=not R1*R2. The G2 gate was designated as CK-FITC-positive, CD45-negative to weakly positive CTCs. According to the location of each cell group, the R2 region was selected using a polygon tool. The R2 region is a pentagon in which the first side (a) shows the CD45-negative to weakly positive cells in R2; the second side (b) parallels the impurities of the specimen to a certain distance to reduce the false-positive results caused by non-specific staining of impurities; the third side (c) shows the CK-FITC-positive cells in R2; and the fourth and fifth sides overlap with the plot frame.

Panels B and C show the results from patient No.3. The final result of the sample is the number of CTCs in the EpCAM-positive section minus the number of CTCs in the inner control. Panel B (left) shows the result of the EpCAM-positive section in the PPB sample from patient No.3. There were 44 CTCs in R2. Panel B (right) shows the inner-control of the PPB sample from patient No.3 there were no CTCs in R2. Therefore, the final result was 44 CTCs. Panel C (left) shows the result of the EpCAM-positive section in the IPVB sample from patient No.3. There were 524 CTCs in R2. Panel C (right)

shows the inner-control of the IPVB sample from patient No.3. There were 4 CTCs in R2. Therefore, the final result was 520 CTCs.

Abbreviations: CTCs: circulating tumor cells; PPB: preoperative peripheral blood; IPVB: intraoperative pulmonary venous blood; G: gate; R: region; A2: LTEP-A2 human lung adenocarcinoma cell line.

Figure S2 Selection of the CTC cutoff point according to the ROC curve

Figure S2 legend

Panel A

AUC (area under the curve)=0.919, p=0.001, 95%CI 0.808-1. First, different cutoff points from 1-45 were acquired, and the sums of the sensitivity and specificity were calculated. The optimum cutoff point corresponding to the largest sum of sensitivity and specificity was selected. For the cutoff point of 4.5, sensitivity + specificity=0.692; for the cutoff point of 5.5, sensitivity + specificity=0.646. These two values were the largest, and thus, we selected >5 CTCs/15 mL as the PPB cutoff point.

Panel B

AUC (area under the curve)=0.888, p=0.002, 95%CI 0.755-1. Different cutoff points from 1-521 were acquired. Then, the sums of the sensitivity and specificity were calculated. The optimum cutoff point corresponding to the largest sum was selected. For the cutoff point of 25.5, sensitivity + specificity=0.746. This was value the largest

value, and thus, we selected >25 CTCs/15 mL as the IPVB cutoff point.

Abbreviations: PPB: preoperative peripheral blood; IPVB: intraoperative pulmonary venous blood;

Figure S3 The selection of CTC cutoff point according to the univariate Cox regression analysis

Figure S3 legend

Panel A

We selected integers between 1-10 as possible cutoff points. The Cox proportional hazards regression analysis shows that for cutoff points of 5, 6, 7, and 8, $p<0.05$; therefore, the cutoff point >5 CTCs/15 mL corresponds to the largest HR of 8.71. The results of the Cox proportional hazards regression analysis also support the PPB CTC cutoff point >5 CTCs/15 mL.

Panel B

We selected integers in multiples of 5 between 10-80 as possible cutoff points. The Cox proportional hazards regression analysis shows that for cutoff points from 20-75, $p<0.05$; therefore, the cutoff point >25 CTCs/15 mL ($p=0.016$) corresponds to the largest HR of 12.88. The results of the Cox proportional hazards regression analysis also support the IPVB CTC cutoff point >25 CTCs/15 mL.

Abbreviations: CTCs: circulating tumor cells; PPB: preoperative peripheral blood;

IPVB: intraoperative pulmonary venous blood; HR: hazard ratio

Table S1. The sensitivity and recovery rate of the FAMCell System for detecting EpCAM-positive cells spiked in leukocytes *

Spiked EpCAM-positive cells†	Detected EpCAM-positive cells	SD	Sensitivity	Recovery %‡
5×10^3	2111.80	197.75	$1/10^4$	42.24
5×10^2	228.40	25.72	$1/10^5$	45.68
5×10^1	21.20	3.35	$1/10^6$	42.40
5×10^0	0		$1/10^7$	0

*Different amounts of EpCAM-positive cells were spiked in 1×107 leukocytes from healthy volunteers. Each experiment was repeated 3 times.

†95.9% of the LTEP-A2 human lung adenocarcinoma cells were identified as EpCAM-positive cells (data not shown).

‡The recovery of EpCAM-positive cells was calculated as the number of detected cells divided by the number of spiked cells.

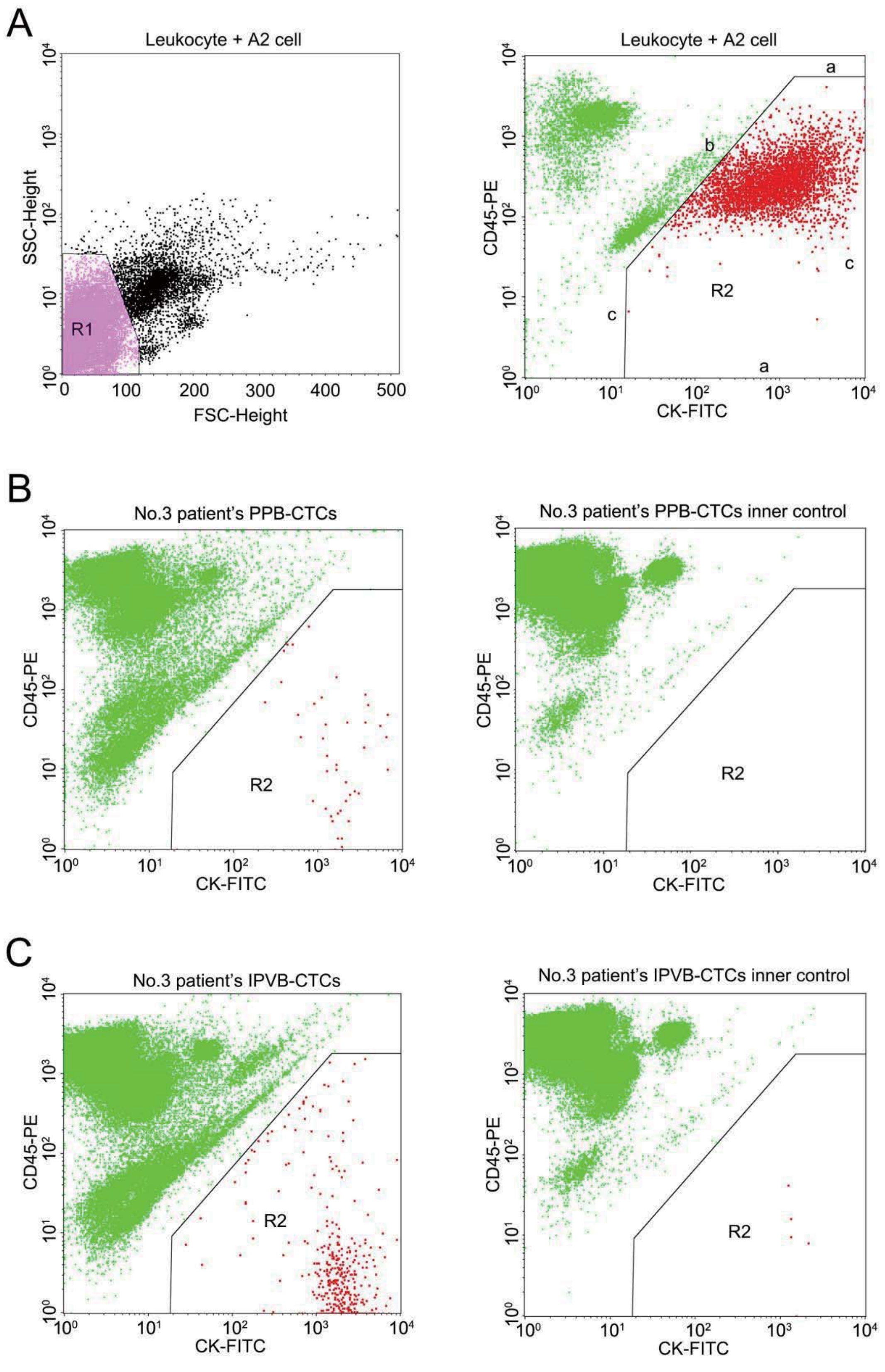
A
Leukocyte + A2 cell
SSC-Height
FSC-Height
R1
Leukocyte + A2 cell
CD45-PE
CK-FITC
R2
a
b
c
B
No.3 patient's PPB-CTCs
No.3 patient's PPB-CTCs inner control
CD45-PE
CK-FITC
R2
C
No.3 patient's IPVB-CTCs
No.3 patient's IPVB-CTCs inner control
CD45-PE
CK-FITC
R2

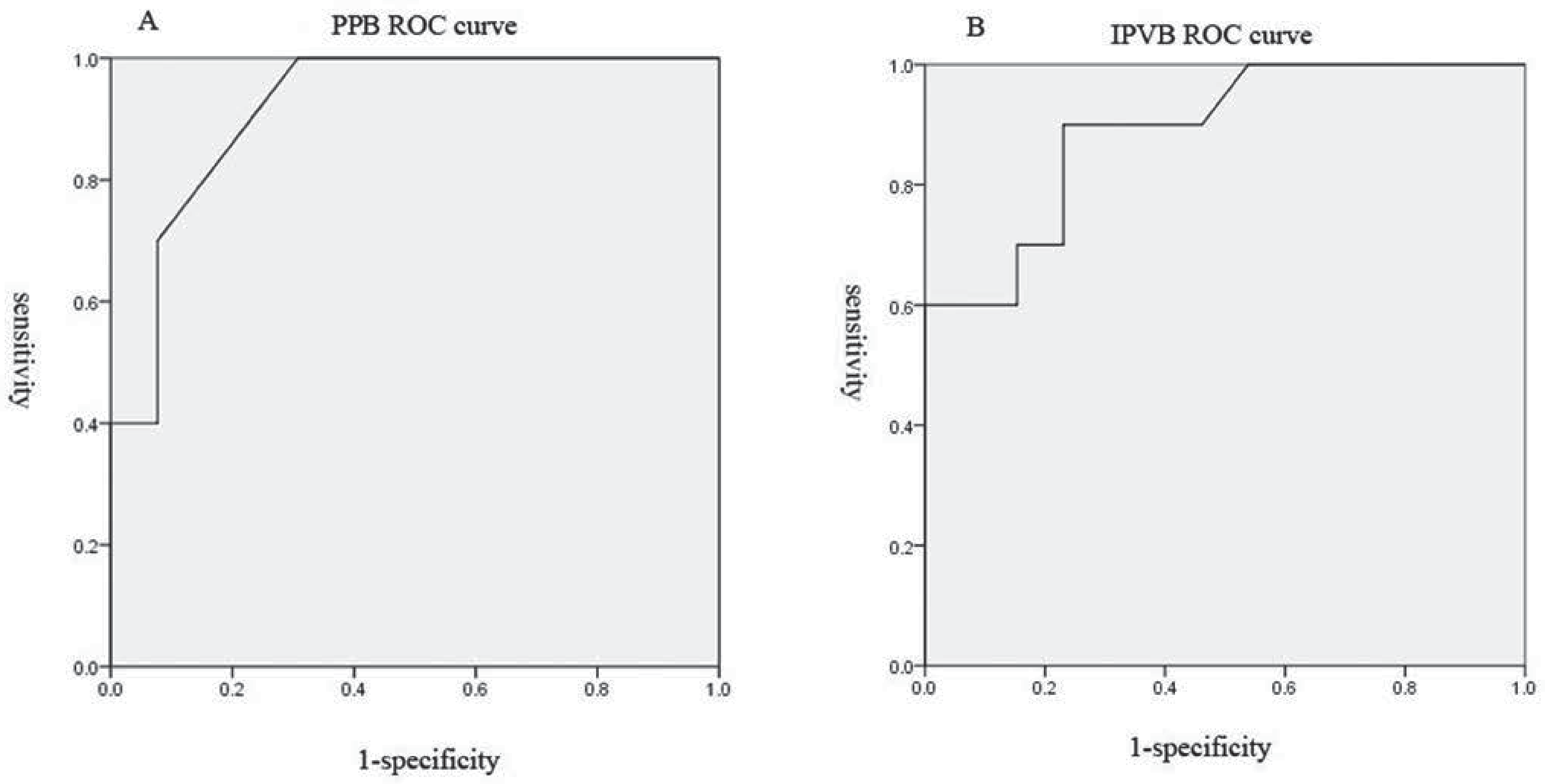
A
PPB ROC curve
sensitivity
1-specificity
0.0
0.2
0.4
0.6
0.8
1.0
B
IPVB ROC curve
sensitivity
1-specificity

A

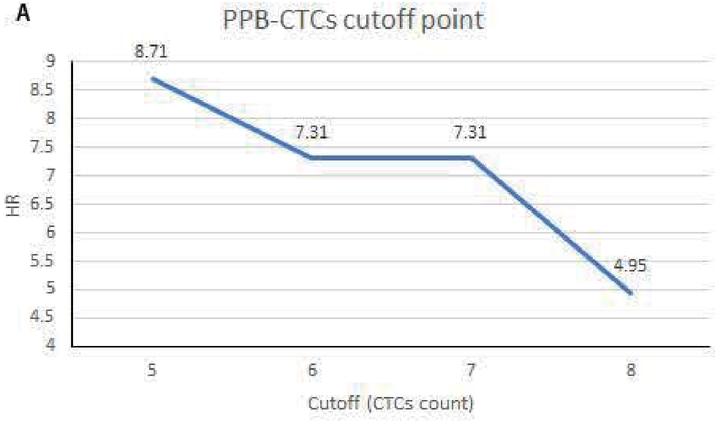
PPB-CTCs cutoff point
HR
Cutoff (CTCs count)
8.71
7.31
7.31
4.95
9
8.5
8
7.5
7
6.5
6
5.5
5
4.5
4
5
6
7
8

B

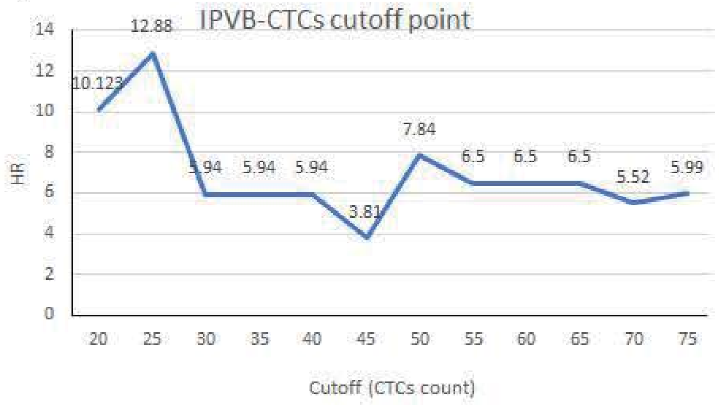
IPVB-CTCs cutoff point
HR
Cutoff (CTCs count)
10.123
12.88
5.94
5.94
5.94
3.81
7.84
6.5
6.5
6.5
5.52
5.99
14
12
10
8
6
4
2
0
20
25
30
35
40
45
50
55
60
65
70
75
